# Enhanced Electromagnetic Wave Absorption Properties of Poly(3,4-ethylenedioxythiophene) Nanofiber-Decorated Graphene Sheets by Non-covalent Interactions

**DOI:** 10.1007/s40820-015-0067-z

**Published:** 2015-10-13

**Authors:** Xiang Zhang, Ying Huang, Panbo Liu

**Affiliations:** Key Laboratory of Space Applied Physics and Chemistry, Ministry of Education, School of Science, Northwestern Polytechnical University, Xi’an, 710129 People’s Republic of China

**Keywords:** Graphene, PEDOT, Nanofibers, Electromagnetic wave absorption

## Abstract

Graphene sheets (GNs) have high conductivity, but they exhibit weak electromagnetic (EM) wave absorption performance. Here, poly (3,4-ethylenedioxythiophene) (PEDOT) nanofibers were decorated on the surface of GNs in which the residual defects and groups act as the active sites and therefore are beneficial for the deposition of PEDOT nanofibers. The SEM images display that PEDOT nanofibers are successfully decorated on the surface of GNs through in situ polymerization. The diameter of the PEDOT nanofibers were ranged from 15 to 50 nm with hundreds of nanometers in length. The EM wave absorption properties of graphene, PEDOT, and GNs-PEDOT were also investigated. Compared to pure graphene and PEDOT, the EM wave absorption properties of GNs-PEDOT improved significantly. The maximum value of *R*_L_ was up to −48.1 dB at 10.5 GHz with a thickness of only 2 mm. Meanwhile, the absorption bandwidth of *R*_L_ values below −10 dB was 9.4 GHz (5.8–12.3, 12.9–15.8 GHz) in the thickness of 1.5–3 mm. The enhancement is attributed to the modification of PEDOT and the unique structure of nanofibers. On one hand, the deposition of PEDOT nanofibers on the surface of GNs decreases the conductivity of graphene, and makes impedance match better. On the other hand, the unique structure of PEDOT nanofibers results in relatively large specific surfaces areas, providing more active sites for reflection and scattering of EM waves. Therefore, our findings demonstrate that the deposition of conducting polymers on GNs by non-covalent bond is an efficient way to fabricate strong EM wave absorbers.

## Introduction

In recent decades, electromagnetic interference (EMI) problem has emerged due to the increasing use of electronic devices and communication facilities in most areas, leading to complex electromagnetic environment. An effective way to solve the problem is developing EM wave absorbing materials, which can absorb EM wave effectively and convert EM energy into thermal energy or dissipate the EM waves by interference. Therefore, considerable efforts have been made to develop strong, lightweight, and broadband absorbing materials [1–3]. Typically, traditional materials such as ferrite, graphite, and carbon nanotubes (CNTs) have been used for EM wave absorption materials due to their high conductivity and good dielectric properties [4–6]. However, these materials cannot reach the standards of ideal EM wave absorption materials. The high density, corrosion susceptibility, and narrow bandwidth of absorption frequency limit ferrite’s applications [7–9]. Similarly, graphite has poor dispensability and relatively low absorbing abilities. Likewise, CNTs are economically non-viable, difficult to produce at bulk scale and require purification, auxiliary treatment, and functionalization steps [10, 11]. Therefore, designing and fabricating new type of EM wave absorption materials are highly demanded.

Graphene, a novel two-dimensional carbon material, has triggered enormous interest due to its fascinating electrical properties [12, 13]. Graphene possesses not only a stable structure but also high-specific surface area and excellent electronic conductivity. These properties make graphene or graphene-based materials very promising as a new type of strong and lightweight EM wave absorption materials [14]. However, the study finds out that the maximum reflection loss of sole GNs is only −6.9 dB, because its good electric conductivity becomes a disadvantageous factor according to the impedance match mechanism [2]. Nevertheless, previous research demonstrates that the residual defects and groups in GNs can act as the active sites, which may be beneficial for grafting (combining) conducting polymers by non-covalent bond, and improving the EM wave absorption properties of GNs. Over the last four decades, conducting polymer-based composites have attracted much attention, because of facile synthesis, light weight, low cost, easy processing ability as well as tunable dielectric and magnetic attributes [15–17]. Among conducting polymers, poly (3,4-ethylenedioxythiophene) (PEDOT) has been considered as a promising candidate for EM wave absorbers due to its tunable dielectric and magnetic attributes and excellent environmental stability [18–20]. Yu et al. synthesized graphene/polyaniline (PANI) nanorod arrays and the sample exhibited a maximum absorption of −45.1 dB with a thickness of 2.5 mm [21]. In our previous work, we synthesized graphene–PANI film, and the results showed the maximum reflection loss was −41.4 dB with a thickness of 2.0 mm [22]. Ni et al. fabricated PEDOT microspheres with a layer thickness of 2 mm, and the maximum absorption is −25 dB at 15.9 GHz [20]. Even higher reflection loss of −30 dB at 9.5 GHz is observed by synthesizing the Fe_3_O_4_-PEDOT microspheres with EDOT/Fe_3_O_4_ ratio of 20 [23]. To the best of our knowledge, the EM wave absorption properties of GNs-PEDOT nanofibers have never been reported.

In this paper, PEDOT nanofibers were directly grown on the surface of GNs by non-covalent interactions through in situ polymerization. The maximum reflection loss of the nanocomposites reaches −48.1 dB, and the absorption bandwidth exceeding −10 dB is 3.1 GHz with a thickness of only 2 mm. Meanwhile, the absorption bandwidth corresponding to reflection loss below −10 dB is 9.4 GHz (5.8–12.3, 12.9–15.8 GHz) in the range of 1.5–3 mm. Moreover, the addition amount of the nanocomposites into the paraffin matrix is only 25 wt%. Thus, GNs-PEDOT nanofibers are very promising as lightweight EM wave-absorbing materials.

## Experimental

### Preparation

Graphene oxide (GO) was prepared by Hummers method [24]. The nanocomposites were synthesized as follows: Firstly, 0.2 g GO was dispersed in 200 mL deionized water and ultrasonicated for 1 h, then 0.1 mL hydrazine was added and heated at 90 °C for 24 h. Secondly, the obtained GNs were dissolved in deionized water by sonication treatment, afterward, 0.2 mL 3,4-ethylenedioxythiophene monomers dispersed in ethanol and 2 mL H_2_SO_4_ were added. Then, (NH_4_)_2_S_2_O_8_, the oxidation, was added in the above solution. Thirdly, the mixture was cooled down to 0 °C and stirred for 24 h. The obtained product was washed with water until pH 7 and dried at 60 °C for 12 h.

### Characterization

The molecular structures of GNs-PEDOT nanofibers were observed on Fourier transform infrared spetctroscopy (FTIR, NICOLET iS10). Raman spectroscopy were characterized by Renishaw in Via Raman Microscope. The chemical states were investigated by X-ray photoelectron spectroscopy (XPS, PHI 5300×). The morphology were characterized by field-emission scanning electron microscope (FESEM, Quanta 600FEG) and transmission electron microscope (TEM, Tecnai F30 G^2^). The electromagnetic parameters of samples were measured in a HP8753D vector network analyzer at the frequency range of 2–18 GHz. The samples were prepared by mixing 25 wt% of the nanocomposites with a paraffin matrix uniformly. The mixture was then pressed into toroidal-shaped samples (*H* 2.0 mm, *φ*_out_ 7.0 mm, *φ*_in_ 3.0 mm). The input power level of the incident microwave is −5.0 dBm.

## Results and Discussion

**Fig. 1:**
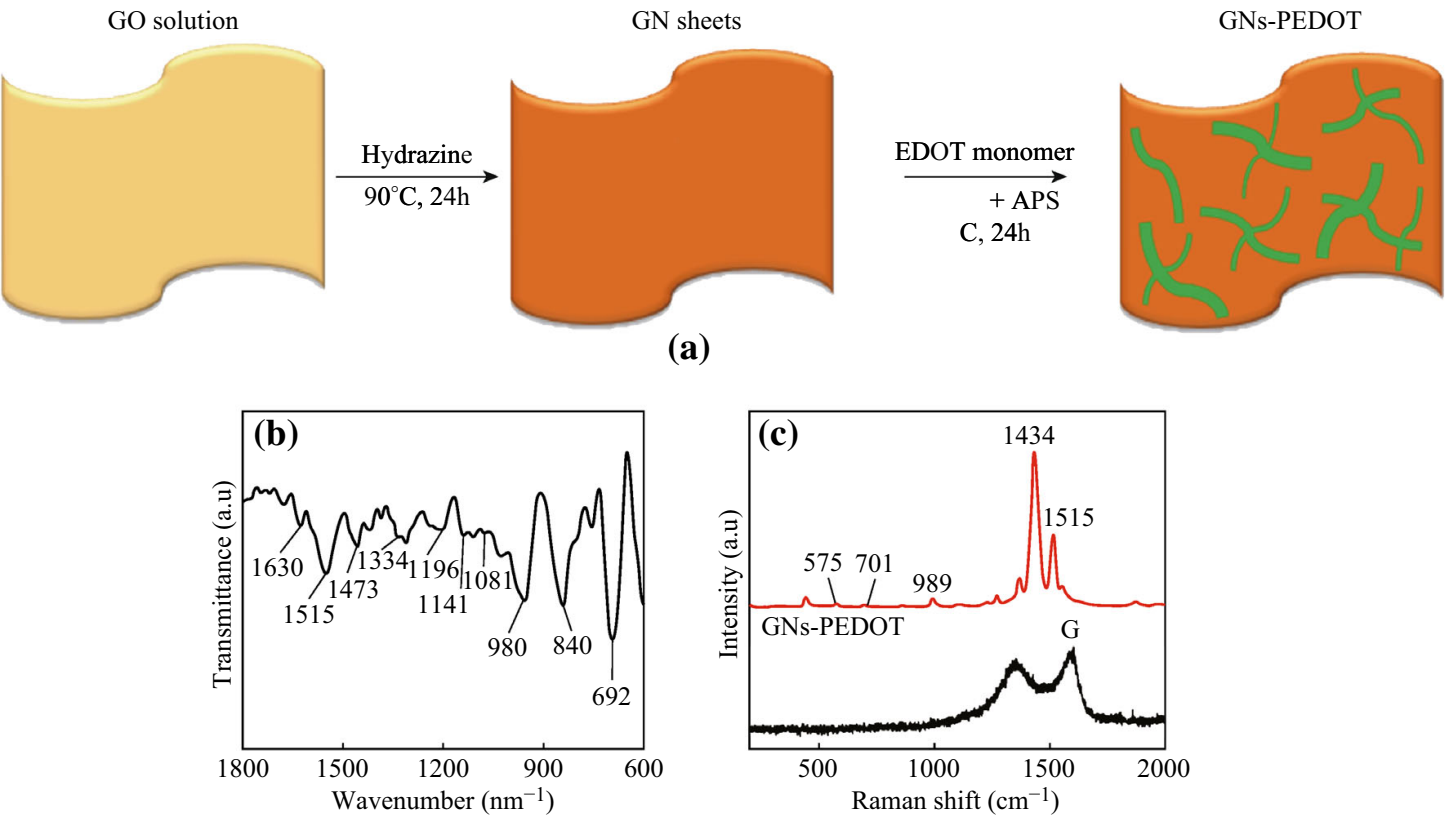
**a** Schematic illustration of GNs-PEDOT. **b** FTIR spectra of GNs-PEDOT. **c** Raman spectra of GO and GNs-PEDOT

The formation mechanism of GNs-PEDOT is schematically depicted in Fig. 1a. GO was firstly reduced to GNs by hydrazine. Then, 3,4-ethylenedioxythiophene (EDOT) monomers were grafted on the active sites (residual defects and groups in GNs) by the electrostatic attraction. With the addition of H_2_SO_4_ and (NH_4_)_2_S_2_O_8_, EDOT monomers began to polymerize; thus, PEDOT nanofibers were formed. Figure 1b shows the FTIR spectra of GNs-PEDOT. The characteristic peaks at 1515 and 1334 cm^−1^ are attributed to the C=C/C–C stretching vibrations of the thiophene rings. The peaks at 1196, 1141, and 1081 cm^−1^ are ascribed to the C–O–C stretching, and the bands at 980, 840, and 692 cm^−1^ are ascribed to the C–S bond in the thiophene rings, respectively. The results indicate that PEDOT is successfully grafted on the surface of GNs [25]. Figure 1c shows the Raman spectra of GO and GNs-PEDOT. For GO, the Raman spectrum is associated with a typical D band at 1350 cm^−1^ and a G band at 1590 cm^−1^. For GNs-PEDOT, apart from the D and G bands, the bands at 1434 and 1515 cm^−1^ are attributed to C=C stretching, whereas the bands at 575 and 989 cm^−1^ are assigned to oxyethylene ring deformation, and the band at 701 cm^−1^ is related to symmetric C–S–C deformation, suggesting the formation of PEDOT [26]. In addition, the D and G bands in GNs-PEDOT exhibit a blue or red shift compared with GO due to the strong interactions between GNs and PEDOT.

**Fig. 2:**
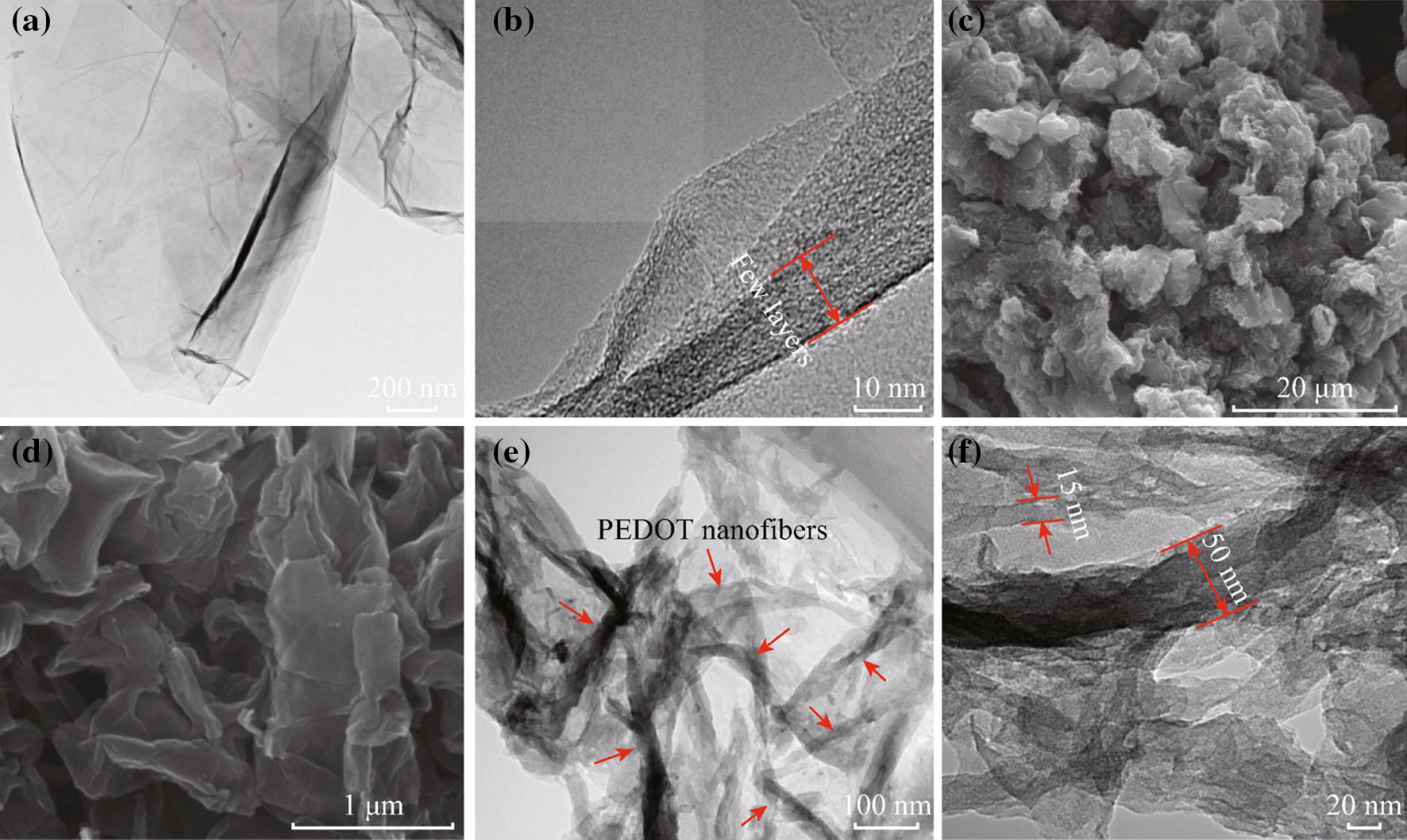
**a, b** TEM images of GNs. **c, d** FESEM images of GNs-PEDOT. **e, f** TEM images of GNs-PEDOT

The morphology of the samples are shown in Fig. 2. In Fig. 2a, GNs exhibit typical transparent and rippled silk morphology. The HRTEM image of GNs signifies that GNs appear flat except for some wrinkles at the edge of it. The FESEM images in Fig. 2c, d show that GNs look like crumpled and curved attributing to the PEDOT nanofibers coated on the surface. Figure 2e displays the TEM image of GNs-PEDOT. It can be clearly seen that the surface of GNs is homogeneously covered with PEDOT nanofibers, and the PEDOT nanofibers distribute on the surface of GNs as indicated by the red arrows. Meanwhile, no free PEDOT nanofibers or naked GNs were observed, indicating that the nucleation and growth of PEDOT nanofibers only occur on the surface of GNs. Figure 2f shows the enlarged images of GNs-PEDOT. It can be clearly observed that the diameter of the PEDOT nanofibers ranges from 15 to 50 nm with hundreds of nanometers in length, which is different from previous report [16]. The special morphology may be favorable for the enhancement of the charge transfer between GNs and PEDOT nanofibers.

**Fig. 3:**
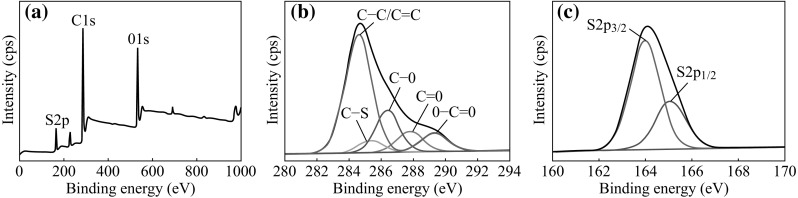
**a** XPS spectrum, **b** C 1*s* and **c** S 2*p* spectra of GNs-PEDOT

XPS was used to verify the surface composition of GNs-PEDOT and the results are presented in Fig. 3. The wide scan XPS spectrum in Fig. 3a indicates that the existence of C, O, S elements in the nanocomposites, and no other elemental signals were detected in the general XPS spectrum. Figure 3b shows the C1 s spectra of GNs-PEDOT which is deconvoluted into five different peaks. The peaks centered at 284.6, 286.4, 287.8, and 289.3 eV may be, respectively, attributed to C–C/C=C, C–O or alkoxy, C=O, and O–C=O groups. In addition, the new peak centered at 285.3 eV is for the C–S group [27]. The S 2*p* XPS spectra (see Fig. 3c) show the presence of sulfur spin-split doublet at around 164.0 eV (S 2*p*_3/2_) and 165.1 eV (S 2*p*_1/2_) with an energy splitting of 1.1 eV, suggesting the formation of doped PEDOT.

**Fig. 4:**
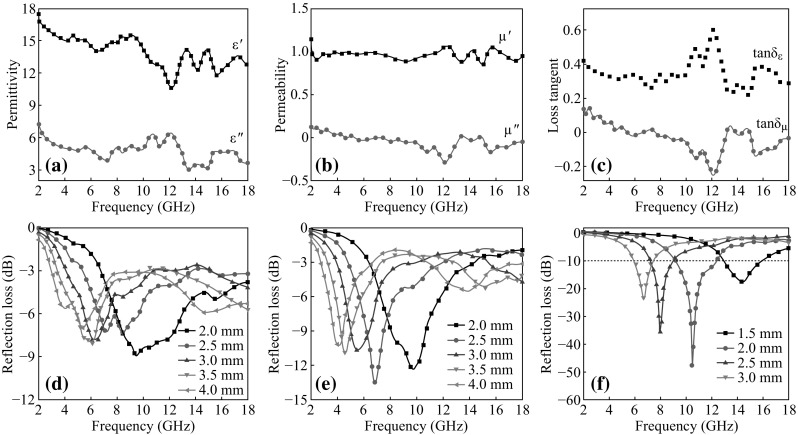
The complex relative permittivity (**a**), permeability (**b**), and loss tangent (**c**) of GNs-PEDOT. Reflection loss curves of **d** GNs, **e** PEDOT and **f** GNs-PEDOT

To understand the EM wave absorption mechanisms, the real part (*ε*′) and imaginary part (*ε*′′) of the relative complex permittivity, as well as the real part (*µ*′) and imaginary part (*µ*′′) of the relative complex permeability of GNs-PEDOT were investigated. In Fig. 4a, it can be seen that the *ε*′ values varied from 17.5 to 10.8, and the ε′′ values decrease from 7.3 to 3.6 with several fluctuations in the frequency range of 2–18 GHz. In Fig. 4b, it reveals that the *µ*′ values are in the range of 0.9–1.1 and the *µ*′′ values are less than 0.2 over 2–18 GHz. The dielectric loss tangent (tan*δ*_ε_ = *ε*′′/*ε*′) and magnetic loss tangent (tan*δ*_µ_ = *µ*′′/*µ*′) are shown in Fig. 4c. It can be noted that the tan*δ*_ε_ values are higher than tan*δ*_µ_ in the range of 2–18 GHz, suggesting that GNs-PEDOT mainly depends on the dielectric loss.

To clarify the EM absorption properties, the reflection losses (*R*_L_) were calculated according to formula (1) and (2),

1 $$R_{L} \left( {\text{dB}} \right) = 20\log \left| {\frac{{Z_{\text{in}} - 1}}{{Z_{\text{in}} + 1}}} \right|,$$

2 $$Z_{\text{in}} = \sqrt {\mu_{r} /\varepsilon_{r} } \tanh \left[ {j\left( {2\pi fd/c} \right)\sqrt {\varepsilon_{\text{r}} \mu_{\text{r}} } } \right],$$

where *Z*_in_ is the input impedance of the absorber, *c* the velocity of electromagnetic waves in free space, *f* the frequency, and *d* the layer thickness.

In Fig. 4d and e, one can clearly see that pure GN exhibits poor EM wave absorption properties in the range of 2–4 mm, and the maximum *R*_L_ is only −8.9 dB at 9.5 GHz. Similarly, pure PEDOT has weak attenuation to EM wave, and the maximum *R*_L_ is only −14.5 dB at 7 GHz. After coating PEDOT nanofibers on the surface of GNs, the GNs-PEDOT nanofibers show better EM wave absorbing properties in terms of both maximum *R*_L_ value and absorption bandwidth. The maximum *R*_L_ is up to −48.1 dB at 10.5 GHz, and the absorption bandwidth corresponding to the *R*_L_ values below −10 dB is 3.1 GHz (from 9.2 to 12.3 GHz) with a thickness of 2 mm. Furthermore, it is obvious that the maximum *R*_L_ values are less than −17 dB with the thickness of 1.5–3 mm. The results also demonstrate that GNs-PEDOT nanofibers present better EM wave absorption properties than PEDOT microspheres [20], flake-like PANIs [28] and graphene/PANI nanorods [21]. The enhancement is attributed to the modification of PEDOT nanofibers and the unique structure. On one hand, the deposition of PEDOT nanofibers on the surface of GNs decreases the conductivity of graphene, and makes impedance match better. On the other hand, the unique structure of PEDOT nanofibers results in relatively large specific surfaces areas, providing more active sites for reflection and scattering of EM waves. In addition, the reflection loss is sensitive to the heteroatom contents. Due to the heterogeneous media, there are more remarkable interfacial polarization and electron polarization which are helpful to improve the EM wave absorption performance. Furthermore, since PEDOT nanofibers with high conductivity grew on GNs by non-covalent bond, the charge transfer between GNs and PEDOT nanofibers contributes to the excellent EM wave absorption performance as well [21].

## Conclusions

In summary, GNs-PEDOT nanofibers were synthesized via in situ polymerization method, and their EM wave absorption properties were investigated. TEM images indicate that PEDOT nanofibers were grafted on GNs, and the diameter ranged from 15 to 50 nm. The results state that poor EM absorption properties of GNs can be significantly improved by deposition of PEDOT nanofibers on the surface of the GNs. The maximum reflection loss of GNs-PEDOT is −48.1 dB with a thickness of only 2 mm, and the absorption bandwidth corresponding to reflection loss below −10 dB is 9.4 GHz (5.8–12.3, 12.9–15.8 GHz) in the thickness of 1.5–3 mm. Thus, the deposition of dielectric nanostructures on the surface of the GNs is an efficient way to fabricate lightweight materials for strong and lightweight EM absorbers.
